# Using ChatGPT in family medicine and primary health care

**DOI:** 10.4102/safp.v66i1.5895

**Published:** 2024-03-19

**Authors:** Indiran Govender, John Tumbo, Sailesh Mahadeo

**Affiliations:** 1 Department of Family Medicine and Primary Health Care, School of Medicine, Sefako Makgatho Healthcare Sciences University, Pretoria, South Africa; 2 Emergency Medicine, Harry Gwala Regional Hospital, Pietermaritzburg, South Africa

Artificial intelligence (AI) and its influence on medical practice, medical education and research is very topical and is influencing new policies at many institutions including universities and medical journals. Its influence is and will be dramatic and we cannot avoid discussing and using AI. In this article, we present what it can do and provide a platform for more conversations on the use of Chat Generative Pretrained Transformer (ChatGPT) in family medicine and primary health care.

Chat Generative Pretrained Transformer is a language-based AI chatbot launched by the information technology developer OpenAI in November 2022.^1^ It is an interactive machine learning model that utilises conversations with users to gain insight into information used to pretrain the system and automate tasks. The model enables users to hold conversations with the system and steer the discussion towards desired details and outputs. The ChatGPT is designed to create convergence between human and AI in a wide variety of circumstances including health, finance, banking, security, academia, research, information technology, communication, judiciary and education.^2^

To demonstrate the general capabilities of AI, the following question was posed to ChatGPT as an example of postgraduate assignment in family medicine and primary health care: *Evaluate the evidence regarding the use of Direct Oral Anticoagulants (DOACs) in family practice.* The ChatGPT brought up the following response within 5 seconds:^3^

- Direct Oral Anticoagulants (DOACs) have been extensively studied and have demonstrated efficacy and safety in various clinical scenarios. DOACs are a newer class of anticoagulant medications that includes drugs like dabigatran, rivaroxaban, apixaban, and edoxaban. Here is an overview of the evidence supporting the use of DOACs:
- Stroke Prevention in Atrial Fibrillation (AF): Multiple large-scale clinical trials, such as the RE-LY trial for dabigatran, ROCKET AF for rivaroxaban, ARISTOTLE for apixaban, and ENGAGE AF-TIMI 48 for edoxaban, have shown that DOACs are at least as effective as warfarin in preventing stroke and systemic embolism in patients with non-valvular atrial fibrillation.
- Venous Thromboembolism (VTE) Treatment and Prevention: DOACs have been proven to be non-inferior or superior to conventional anticoagulation (such as warfarin) in the treatment of acute VTE.
- Secondary Prevention of Cardiovascular Events: DOACs have shown efficacy in preventing recurrent cardiovascular events in patients with a history of acute coronary syndrome (ACS) or stable coronary artery disease.
- Thromboprophylaxis in Medically Ill Patients: Some DOACs have demonstrated effectiveness in preventing venous thromboembolism in acutely ill medical patients during and after hospitalization.
- Safety and Predictability: DOACs have a more predictable anticoagulant effect than warfarin and have a lower risk of drug and food interactions compared to warfarin.
- It’s important to note that while DOACs offer advantages, they may not be suitable for all patients, and individualized treatment decisions should be made based on factors such as patient characteristics, comorbidities, and specific clinical scenarios. As medical guidelines are periodically updated, consulting the most recent guidelines and literature is advisable for the latest information.

The use of ChatGPT in clinical practice as in family medicine and primary care has relevance in the following aspects^4^:

1. Diagnostic assistance: AI algorithms can analyse medical images, such as X-rays, magnetic resonance imaging (MRIs) and computed tomography (CT) scans, to assist healthcare professionals in diagnosing conditions. Artificial intelligence can help detect abnormalities, tumours and other anomalies more quickly and accurately than traditional methods.
2. Predictive analytics: AI can analyse patients’ data to identify patterns and predict potential health issues and can be particularly valuable in managing chronic conditions, predicting disease progression and determining optimal treatment plans.
3. Clinical decision support systems (CDSS): AI-powered CDSS integrates patients’ data, medical literature and best practices to assist in diagnosis, treatment planning and provide healthcare professionals with real-time information and evidence-based recommendations to support clinical decision-making.
4. Personalised medicine: AI is instrumental in advancing personalised medicine by analysing genetic data, patients’ history and other relevant information to tailor treatment plans based on an individual’s unique characteristics.
5. Remote patient monitoring: AI technologies enable continuous monitoring of patients outside traditional healthcare settings. Wearable devices and sensors can collect real-time data, providing healthcare providers with valuable insights into a patient’s health status and enabling early intervention.
6. Natural language processing (NLP): NLP allows computers to understand and process human language. In healthcare, NLP is used for tasks such as extracting information from medical records, improving documentation efficiency and facilitating communication between healthcare professionals.
7. Robotics in surgery: AI-powered robotic systems assist surgeons in performing minimally invasive surgeries with increased precision. These systems can enhance the surgeon’s capabilities and improve patient outcomes.
8. Drug discovery and development: AI accelerates the drug discovery process by analysing vast datasets to identify potential drug candidates, predict their effectiveness and streamline the development process. This has the potential to bring new treatments to market more quickly.
9. Administrative efficiency: AI applications can help streamline administrative tasks in healthcare facilities, such as appointment scheduling, billing and electronic health record (EHR) management. This efficiency allows healthcare professionals to focus more on patient care.
10. Patient engagement: AI technologies can contribute to patient engagement by providing personalised health information, reminders for medication and appointments and virtual health assistants that answer queries and offer guidance.

Clearly, ChatGPT has the capabilities to capture the essence of a conversation accurately, efficiently and produce very detailed responses. It provides data that users could employ directly to the scenario or subject it to review and modification to customise it to the need. The use of AI in the clinical setting leverages on the strength of both human expertise and AI for enhanced medical diagnosis, treatment planning and overall patient care.^5^ These advances have the potential to revolutionise clinical care by improving efficiency, accuracy, effectiveness of therapies and overall patient outcomes.

The information can, however, be inadvertently used and even presented as the user’s response to the assignment. Similarly, researchers may opt to use ChatGPT to write manuscripts for publication by loading in the data instead of actively interacting with the data, interpreting it and discussing the implications the traditional way. This demonstrates the potential misuse of ChatGPT in the academic setting and clearly calls for stringent measures to restrict access of the ChatGPT to primary data and research publications in order to maintain academic credibility and authenticity.

Another pitfall of ChatGPT is the potential it could have to change traditional interactive and active clinical methods of diagnosis to one where the practitioner enters all data presented by the patient into the computer system, which uses its algorithms and prediction to bring out a diagnosis. The patient may be completely unaware of the fact that the practitioner got the diagnosis from an AI model rather than from the use of interactive clinical reasoning. This may result in the critical absence of the human professional touch in the art of clinical practice.

While ChatGPT and other conversational agents have the potential to transform medical education and research writing by offering personalised, interactive and readily available learning experiences, it is crucial to address challenges such as data privacy, ethical considerations and the need for proper validation and regulation to ensure the safe and responsible integration of AI in healthcare settings. However, while ChatGPT is a powerful language generation tool, it cannot replace human intelligence or replicate the complexity of human thinking. It is prudent to call for cautious use of ChatGPT in Family Medicine cognisant of the potential benefits on the one hand versus the immense harm that may result from unregulated and unvalidated advice.
